# Synergistic Effect of Static Magnetic Field and Modified Atmosphere Packaging in Controlling Blown Pack Spoilage in Meatballs

**DOI:** 10.3390/foods11101374

**Published:** 2022-05-10

**Authors:** Yongfang Chen, Anthony Pius Bassey, Yun Bai, Shuang Teng, Guanghong Zhou, Keping Ye

**Affiliations:** National Center of Meat Quality and Safety Control, Jiangsu Collaborative Innovation Center of Meat Production and Processing, College of Food Science and Technology, Nanjing Agricultural University, Nanjing 210095, China; 15050521228@163.com (Y.C.); bassey_ap44@outlook.com (A.P.B.); baiyun@njau.edu.cn (Y.B.); tengshuang@njau.edu.cn (S.T.); guanghong.zhou@hotmail.com (G.Z.)

**Keywords:** blown pack spoilage, meatballs, *Klebsiella pneumoniae*, static magnetic field, modified atmosphere packaging

## Abstract

This study aimed to compare the microbial diversity in meatballs with or without blown pack spoilage (BPS) to determine the cause of BPS and to assess the synergistic effect of static magnetic field (SMF) and modified atmosphere packaging (MAP) to reduce the phenomenon of BPS. Results showed that the BPS group with a 2.26-fold larger volume and packaging containing 71.85% CO_2_ had *Klebsiella* spp. (46.05%) and *Escherichia* spp. (39.96%) as the dominant bacteria, which was different from the spoilage group. The results of isolation and identification of strains from the BPS group and their inoculation test confirmed that *Klebsiella pneumoniae* was the major strain-inducing BPS in meatballs due to its pack-swelling ability. SMF (5 mT) treatment combined with MAP (40%CO_2_ + 60%N_2_), which did not influence the sensory quality of meatballs, had a significant synergistic effect on preventing the increase in pack volume. Compared with the control group, this synergistic treatment effectively delayed bacterial growth, drop in pH, and the increase of TBARS. The findings of this study will provide further guidance for meatball manufacturers to adopt effective strategies to reduce the BPS of meatballs.

## 1. Introduction

Blown pack spoilage (BPS) is characterized by an increase in gas production, which results in severe distention of the pack [1]. BPS often makes meat and its derivatives undesirable to consumers from an organoleptic standpoint and has been reported in several countries around the world [2,3,4,5,6], instituting a huge challenge to the meat industry globally. Hence, taking effective measures to control its occurrence can reduce economic losses and significantly impact the development of the global meat industry.

BPS is often considered to be relatively induced by the activities of several spoilers. Notably, *Clostridium* (*C.*) spp. is often regarded as the integral contributor of BPS in vacuum-packaged meat, with *C. estertheticum* frequently reported in studies worldwide [7]. Currently, BPS studies are mainly focused on the studies of vacuum-packaged raw meat [2,3,4,5,6] and fermented meat products [8]. Pinheiro et al. [9] also reported that BPS occurrence in cooked meats and lactic acid bacteria (LAB) isolates from cooked pork ham were the cause of blown packs.

Inhibition strategies of BPS have been applied and mainly rely on reducing contamination using sporicidal agents, such as peroxyacetic acid. Broda et al. [10] found that peroxyacetic acid (POAA) sanitizer was able to inactivate at least 4 log CFU/mL *C. estertheticum* spores in vitro. However, treatment with a POAA-based rinse did not demonstrate any significant effect on the mean time to the onset of gas production in the meat model due to the attachment of clostridial spores to the connective or fat tissues of dressed carcasses, indicating the inefficacy of the POAA-based rinse [11]. Moreover, a sporicidal agent such as peroxyacetic acid is highly corrosive and often unaccepted by consumers. Moreover, some countries do not accept red meat that has undergone any chemical treatment [12]. Nonetheless, the agent was more suitable for fresh carcasses and not for cooked meat products as it could alter the overall flavor of the final products. Therefore, it is necessary to find appropriate and effective measures that will not negatively impact the sensory traits of the meat products. 

In recent years, static magnetic field (SMF), a non-contaminating and non-residue physical method, has been remarkable in inhibiting microbial growth by affecting oxidative mechanisms in organisms, biological heat and mass transfer processes, and changing the permeability of cell membranes [13,14]. Studies have shown that low-density constant magnetic fields could inhibit the activity of *Escherichia coli* and damage its cell wall [15,16]. Additionally, modified atmosphere packaging (MAP) could hinder the growth of microorganisms by reducing the activity of microorganisms inside the package and delaying the chemical reaction [17]. Compared to traditional thermal treatment, the above two techniques have the potential to inhibit the activity and proliferation of microorganisms without impacting food flavor, taste, and texture. However, little is known about the potential synergetic effect of SMF and MAP on inhibiting microbial gas production and pack expansion. 

Therefore, this study aimed to determine the cause of BPS in meatballs and then preliminarily assess the synergistic effect of SMF combined with MAP on BPS in meatballs. The findings will further guide meat industries to take adequate measures in controlling BPS.

## 2. Materials and Methods

### 2.1. Meatballs Samples

A total of 50 aerobic-packaged meatballs, constituting a specific proportion of pork ham, back fat, seasonings (sugar, salt, chicken essence, vegetable oil, soy sauce, and cooking wine), additives (sodium tripolyphosphate, sodium hexametaphosphate, and carrageenan), and spices (green onions and ginger), were obtained from a local meat processing outlet. Each pack contained 2 meatballs (50 g each) and marinade. Every sample was placed in chilled insulated boxes and conveyed to the laboratory within 3 h.

### 2.2. Experimental Design

#### 2.2.1. Microbial Comparison in Meatballs with or without BPS

All the samples were stored at 15 °C, and each pack volume was measured daily. When the packaging volume of the BPS group did not change the next day, the meatballs with a double increase in the initial volume were classified as the BPS group, while the other samples were classified as the spoilage group. The gas compositions and bacterial communities in the BPS and spoilage groups were investigated to provide insight into the gas changes and the associated microbial consortium. 

#### 2.2.2. Screening and Characteristics of Gas-Forming Bacterial Strains in BPS Meatballs

The bacteria were screened out through traditional plate culture, and each pure colony was inoculated into tryptic soy broth (TSB) containing an inverted Durham tube. The isolates, which produced bubbles during the liquid culture, were confirmed as gas-producing bacteria. Subsequently, the gas-producing strains were identified by 16S rRNA gene sequences, amplified using the universal primers 27-F (AGAGTTTGATCCTGGCTCAG) and 1492-R (GGTTACCTTGTTACGACTT-3). The PCR products were purified and sequenced by Biozeron Biotechnology Co., Ltd., (Shanghai, China). Furthermore, physiological and biochemical identification of gas-producing strains after inoculation were performed. The test was performed using Biochemical tubules (Haibo Co., Ltd., Qingdao, China) as outlined by the manufacturer, including Gram stain, Oxidase, hydrogen sulfide (H_2_S), Ornithine decarboxylase, Methyl red (MR)-Voges–Proskauer (VP) test, Urease, Citrate utilization, Malonate utilization, Inositol fermentation, Glucose fermentation, and Rhamnose fermentation.

#### 2.2.3. In Situ Inoculation Experiment, Growth and Gas Production Capacity of Gas-Producing Bacteria

The meatballs were treated with irradiation (8 KGy dose) via the ^60^Co source at Hangyu Irradiation Technology Co. Ltd. (Nanjing, China) to eliminate background bacteria. From the results of the bacterial community, *Escherichia* spp. and *Klebsiella* spp. isolates, identified as the predominant species in the BPS group, were chosen and inoculated into treated meatballs with an inoculum of approximately 10^4^ CFU/g. Uninoculated meatballs were denoted as the control group. The sealed packs were stored at 15 °C for 4 d, followed by the measurement of pack volume and gas compositions. After inoculation experiments, the strains that induced pack swelling were confirmed as the main BPS contributors. Notably, the strain with the highest contribution was selected for the subsequent study by evaluating its growth and gas production capacity.

#### 2.2.4. Effects of SMF Combined with MAP on BPS of Meatballs

SMF (5 mT) and MAP (40%CO_2_ + 60N_2_) were integrated to assess their synergistic effect. The meatballs inoculated with gas-producing bacteria were treated as follows: (1) stored in a common incubator after aerobic packaging (control group); (2) stored in a magnetic field incubator set at 5 mT after aerobic packaging (SMF group); (3) stored in an incubator after modified atmosphere packaging (40% CO_2_ + 60% N_2_) (MAP group); (4) stored in a magnetic field incubator (5 mT) after MAP (40% CO_2_ + 60% N_2_) (S + M group). The groups were stored at 15 °C and sampled at 0, 16, 32, 48, 64, and 80 h to evaluate relevant indicators (pack volumes, gas compositions, bacterial enumeration, and pH). Nevertheless, TBARS, TVB-N, and sensory analyses were performed at 0, 16, 48, and 80 h. These analyses were performed to assess the synergistic effect of SMF and MAP on meatballs’ BPS.

### 2.3. Test Methods

#### 2.3.1. Determining Packs Volumes and Gas Compositions

The pack volumes of the meatballs were measured using the water-displacement method described by Li et al. [18], with appropriate modifications. Firstly, water (3 L) was added to a circular beaker. Then, each pack was placed inside, and the height of the liquid level rise (h) was recorded. The pack volume (cm^3^) was calculated using the following equation: V = 3.14 × r × r × h (r = beaker radius). Furthermore, the gas mixtures of the packs were determined using an Oxybaby 6.0i gas analyzer (Witt-Gasetechnik GmbH & Co. KG, Witten, Germany) when BPS occurred. A probe of this gas analyzer was inserted into the packs, and the relative percentages displayed on the screen were noted. 

#### 2.3.2. Enumeration of Microorganisms

Each sample (25 g) was weighed and aseptically transferred into sterile stomacher bags containing 225 mL of sterile saline and thoroughly mixed for 2 min using a stomacher (Bag Mixer 8400 V W, Interscience Co., Bretesche, France). After serial 10-fold dilution, 1 mL of the suspension was spread on a medium (plate count agar, Luqiao Co., Ltd., Beijing, China) and incubated (24 h, 37 °C). The results (*n* = 3) are expressed as log CFU/g.

#### 2.3.3. Bacterial Diversity

The bacterial diversity of samples was performed via high-throughput sequencing (HTS). The bacterial DNA in samples was extracted using a TIAN amp DNA Kit (Beijing Tiangen Co., Ltd., Beijing, China), while the concentration and purity were determined using a NanoDrop 1000 spectrophotometer (Thermo Fisher Scientific, Waltham, MA, USA). The V3-V4 region of 16S rRNA genes was amplified by PCR using a range of universal primers (341F and 806R) with barcode sequences for multiplexing reads of each sample. Illumina PE250 library construction and sequencing were carried out according to Li et al. [18]. After the samples were distinguished, the OTU cluster analysis and species taxonomy analysis, including principal component analysis (PCA) and bacterial community, were performed. 

#### 2.3.4. Growth Curves of Gas-Forming Bacterial Strains

Each gas-forming isolate was inoculated in a cell culture plate, and an automatic growth curve analyzer (Oy Growth Curves Ab Ltd., Helsingfors, Finland) was used to measure the growth of 35 strains at 15 °C. Growth curves were fitted by the Huang model (IPMP, 2013) in the USDA Integrated Pathogen Modeling Program (IPMP) 2013 tool [19]. Lag stands for the lag time, and Y_max_ stands for the maximum bacterial count.

#### 2.3.5. pH

Each sample (10 g) was mixed in 90 mL distilled water and thoroughly homogenized (Ultra-Turrax T25, IKA, Berlin, Germany) for 30 s at 6000 rpm. The samples were measured using the pH meter (Hanna 211 pH meter, Hanna, Villafranca Padovana, Italy) after calibration with commercial buffers (pH 4.0, 7.0, and 10.0; Thermo Fisher Scientific, Singapore). 

#### 2.3.6. Total Volatile Basic Nitrogen (TVB-N)

The TVB-N contents in the samples were performed following the China National Food Safety Standard method (GB 5009.228-2016). Briefly, 3 g of the minced sample was homogenized in 30 mL distilled water for 30 min. Subsequently, 10 mL of filtered supernatant was pipetted into the distillation tube. Before analysis (Nitrogen apparatus, K1160, HaiNeng Instruments, Jinan, China), 1 g (each) of magnesium oxide was added to the distillation tube. The results (*n* = 3) were expressed as mg of N per 100 g of meat.

#### 2.3.7. TBARS

The procedure outlined by the China National Food Safety Standard method (GB 5009.181-2016) was adopted to determine the TBARS content in the samples. Each sample (5 g) was homogenized with 25 mL trichloroacetic acid solution for 60 s at 12,000 rpm. After centrifuging (4 °C, 10 min, 1000 rpm), 2 mL of supernatant was added to 2 mL of 20 mM 2-thiobarbituric acid and incubated in a water bath (95 °C, 30 min). After cooling, the absorbance of samples was measured at 532 nm (Spectral Max M2e Multifunctional microplate reader; Molecular Devices, LLC., San Jose, CA, USA). The TBARS content was expressed as the number of mg malondialdehyde (MDA) per kg of samples.

#### 2.3.8. Sensory Evaluation

Sensory evaluation was performed by 8 experienced sensory assessors (4 females and 4 males; aged 23–26 years; mean average of 24 years). They were non-smokers with no prior olfactory dysfunction during the evaluation period. Briefly, meatballs were placed on a white ceramic plate and denoted in random numbers to avoid bias. Each sensory assessment was conducted in the same setting without communication between members. The assessment of the color, appearance, and odor of meatballs was carried out by tactile, visual, and olfactory tests using a 10-point Hedonic scale (Table 1).

### 2.4. Statistical Analysis

The results were statistically analyzed using the SAS version 8.0 software (SAS Institute, Cary, NC, USA) and presented as mean ± standard error. Mean differences were performed using Duncan’s multiple comparison method at *p* = 0.05. Principal component analysis (PCA) was analyzed using the community ecology package (Biozeron Biotechnology Co., Ltd., Shanghai, China). R-forge (Vegan 2.0 package) was used to visualize the loading plot.

## 3. Results and Discussion

### 3.1. Pack Volumes and Gas Compositions

**Figure 1:**
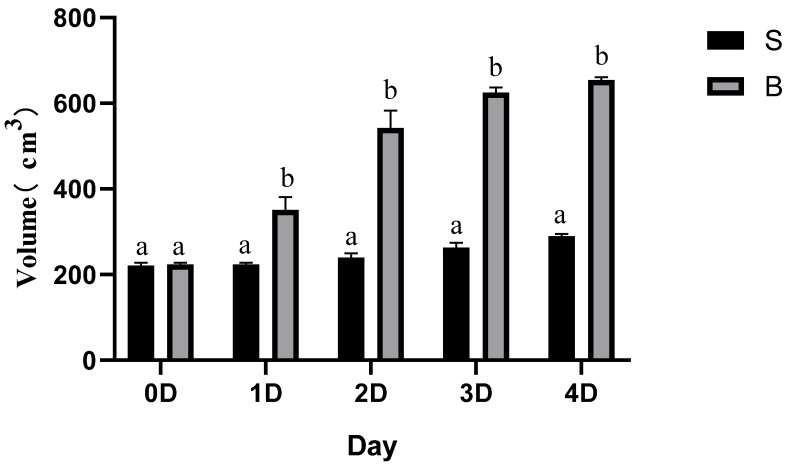
The pack volumes of meatballs during the storage. The different lower cases (a,b) indicate significant differences between the spoilage (S) and BPS (B) groups (*p* < 0.05). The error bars are derived from the standard error of replicates (*n* = 3).

On the fourth day of storage, the percentage of blown packs in all samples was 8.5%, while the pack volumes in the BPS group were 2.26-fold larger than those in the spoilage group (Figure 1). Table 2 showed that the O_2_ composition in the BPS group was 0.13%, against 7.47% in the spoilage group, indicating that O_2_ was almost exhausted in BPS packs. Conversely, CO_2_ composition was significantly higher (*p* < 0.05) in the BPS group (71.85%) than in the spoilage group (7.47%). These findings corroborated the studies of Broda et al. [20], Hernández-Macedo et al. [21], and Li et al. [18], which indicated that CO_2_ dominated the BPS samples.

### 3.2. Microbiological Analysis

As illustrated in Table 2, the bacterial counts showed no significant variation between the BPS and spoilage groups (*p* > 0.05). This revealed that the possible cause of BPS was not attributed to the varying total viable count observed between the meatball groups. 

**Figure 2:**
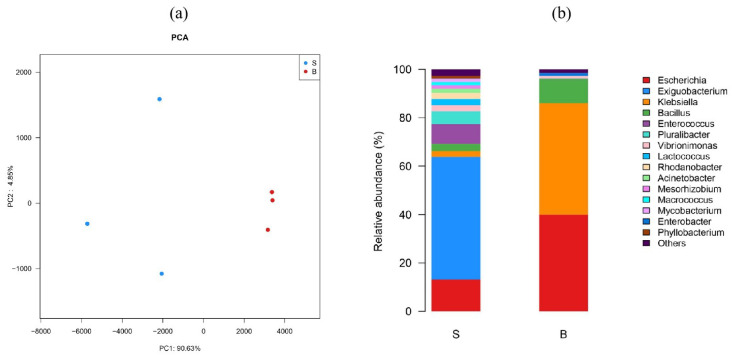
PCA loading plot (**a**) and bacterial diversity (**b**) between the spoilage (S) and BPS (B) meatballs. Every sample was analyzed in triplicate (*n* = 3).

Furthermore, the abundance of the bacterial community between groups was compared by HTS, which could provide a reference for the subsequent screening of gas-producing bacteria. PCA results showed that the bacterial communities of both groups showed a significant separation (Figure 2a), which indicated that the bacterial diversities induced the possibility of BPS. Specifically, the relative abundance (%) at the genus level (Figure 2b) showed that *Exiguobacterium* spp. (50.74%), *Escherichia* spp. (13.13%), and *Enterococcus* spp. (8.18%) were the predominant genera in the spoilage group, while *Klebsiella* spp. (46.05%) and *Escherichia* spp. (39.96%) dominated the BPS group, accounting for > 86% of the total reads. Besides *C. estertheticum* being reported as the main species causing BPS, *Enterobacteriaceae* and LAB have also been linked to BPS occurrences in meat and meat products [18]. Likewise, *Klebsiella pneumoniae* had previously been linked to causing swelling in food [22]. This result supports the hypothesis that *Escherichia* spp. and *Klebsiella* spp. could be involved in causing BPS. 

### 3.3. Isolation and Verification of Package-Swelling Ability of Bacteria Strains in Meatballs

A total of 266 colonies were obtained directly from the spread plate, and Durham-tube tests showed that 133 of these strains could produce gas at 15 °C in vitro. According to the results of bacterial community and gene sequencing, 59 *Klebsiella* spp. and 45 *Escherichia* spp. inoculated into sterile meatballs were used to verify the package-swelling ability in vivo via pack volume determination daily. After storage for 4 d, 35 isolates increased the pack volume to two-fold the initial volume, indicating a high gas production in packs during storage (Table 3). Notably, after molecular biological identification and physiological and biochemical analyses, the 35 isolates were all *K. pneumoniae* [23] (Table 4), which suggested that the bacterium was the primary gas producer in BPS meatballs. 

The growth and gas production capacity of 35 isolates showed that the package volume inoculated with strains C19, C21, and B5 increased obviously among 35 isolates, which increased to 2.74, 2.66, and 2.62 times the initial volume, respectively (Table 3). Additionally, after inoculation with the B5 strain, the CO_2_ content in the pack increased to 70.80%, and the B5 strain showed the shortest lag period and the most significant growth rate (Table 3), which suggested that this isolate had a strong gas production capacity and growth capacity. Therefore, the B5 strain was selected for subsequent experiments to study the inhibition effect of SMF and MAP on BPS.

Although the first report of *K. pneumoniae* causing swelling was in cheese, this is the first study attributing it to BPS in meat products [22]. *K. pneumoniae* is a facultative anaerobic bacterium under *Enterobacteriaceae* that is found in the respiratory and intestinal tracts of humans and animals. It is a common opportunistic pathogen and a food-borne pathogen [24] that induces several infectious diseases in humans, including pneumonia, liver abscesses, septicemia, and diarrhea [25]. Numerous studies have reported its presence in foods, including street foods, vegetables, fish, and meat, in recent years. For example, from 2013 to 2014, Zhang et al. [26] found that the detection rate of *K. pneumoniae* in food was 5.08% (cooked meat products 3.85%, raw meat products 3.87%). Similarly, among 350 ready-to-eat processed meat (luncheon-meat) samples collected in Egypt, 44 (12.6%) *K. pneumoniae* strains were isolated [27]. According to Calbo et al. [28], *K. pneumoniae* induced several foodborne-related outbreaks, with the number increasing gradually. Our findings illustrate that *K. pneumoniae* can also increase the probability of BPS in meat products, indicating its growing threat to the meat industry and public health.

### 3.4. Effects of SMF Combined with MAP on Volume and CO_2_ Content in Packaging

**Figure 3:**
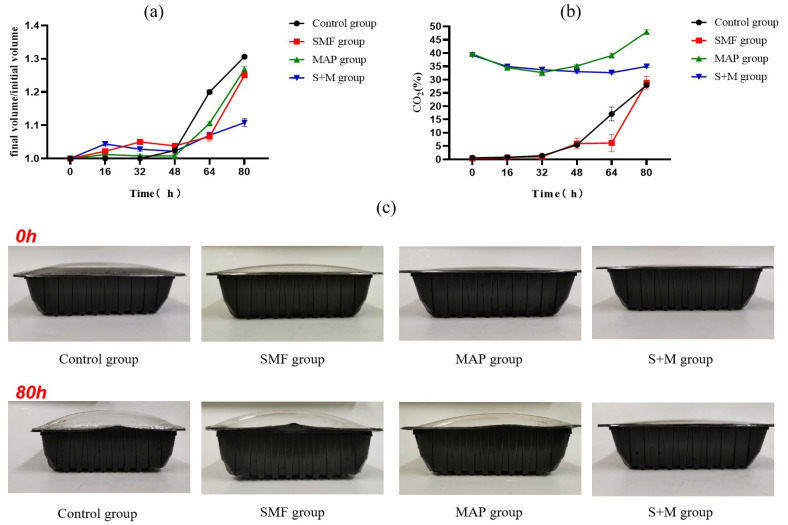
Effects of different treatments on package volume (**a**), CO_2_ content (**b**), and the appearance of the package (**c**). The error bars derived from the standard error of replicates (*n* = 3).

Furthermore, an uptrend in the volume of the control and S + M groups was detected from 32 to 48 h and 48 to 64 h, respectively (Figure 3a). At 80 h, the packs of the control, SMF, and MAP groups were markedly expanded, and the volume change of the S + M group was significantly smaller than that of the other three groups (*p* < 0.05) (Figure 3a,c). These results indicated that SMF combined with MAP treatment could effectively suppress the growth of the packaging volume. Nonetheless, the CO_2_ content in the control, SMF, and MAP groups began to increase from 32 to 48 h, while that of the S + M group was observed from 64 to 80 h (Figure 3b). The CO_2_ ratio of the S + M group was significantly lower than that of the MAP group (*p* < 0.05), indicating the inhibitory effect of SMF + MAP synergy against CO_2_ production. It has been reported that CO_2_ is an essential metabolite produced by microorganisms to break down nutrients such as carbohydrates and sugars [29]. The inhibition of CO_2_ generation by S + M treatment could be attributed to the synergistic influence on bacterial growth or altered metabolic pathways, such as glucose consumption and metabolite production, during bacterial respiration.

The specific intervention available to reduce BPS incidence relies on disinfectants, such as peroxyacetic acid, in the pre-packaging stage of meat [29]. However, Boerema et al. [11] found that treatment with a POAA-based rinse was ineffective in delaying the onset of pack blowing in packs carrying high numbers of *C. estertheticum* spores. Adequate packaging and handling techniques for meat products can be effective against BPS occurrence during product transportation, sale, and storage. MAP technology is widely used in food packaging because its filling gas, CO_2_, can inhibit the ability of bacteria to decompose glucose [30], reduce the generation of metabolites [31], affect bacterial enzyme activity, and reduce growth rates [32]. As a physical processing technology, magnetic fields have also been reported to affect the production of CO_2_ by affecting the metabolic activity of yeast [33]. These demonstrate the potential of MAP and SMF synergy in controlling the metabolic activity of microorganisms and the generation of metabolites. Notably, this is the first study integrating both technologies to elucidate the inhibitory effect against BPS in meat products. 

### 3.5. Effects of SMF Combined with MAP on Bacterial Growth in Meatballs

**Figure 4:**
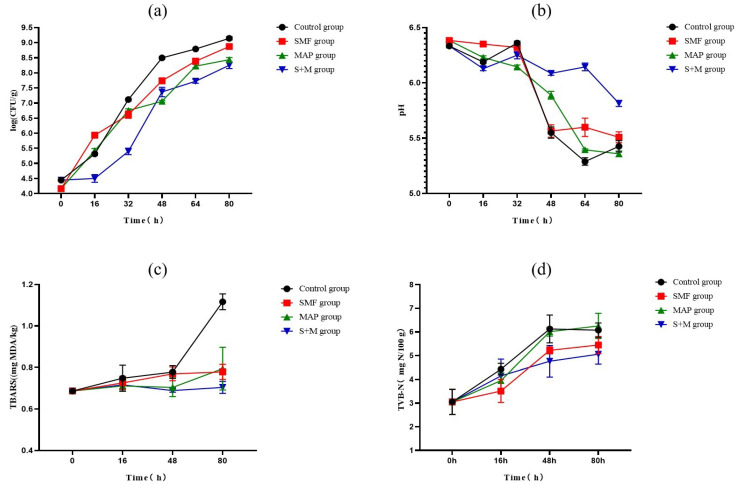
Effects of different treatments on meatballs’ TVC (**a**), pH (**b**), TBARS (**c**), and TVB-N (**d**). The error bars are derived from the standard error of replicates (*n* = 3).

As illustrated in Figure 4a, the gas-producing bacteria of the control, SMF, and MAP groups entered the rapid growth phase at 0–16 h, while the S + M group entered the rapid growth phase at 16–32 h. These phenomena suggested that S + M treatment can delay the entry of bacteria into the logarithmic phase of growth. At 80 h, the number of bacteria in the S + M group was significantly lower than that in the control group (*p* < 0.05). Its inhibitory effect was better than that of the MAP and SMF treatments, indicating that S + M treatment could control the number of microorganisms. According to previous studies, CO_2_ in MAP can inhibit microorganisms, thereby reducing spoilage [34,35]. For example, Guo et al. [36] demonstrated that MAP (40%CO_2_ + 60%N_2_) could effectively inhibit the growth of total viable counts and extend the shelf life of roast chicken meat. Meanwhile, SMF can reduce the metabolic activity of microorganisms and inhibit their growth [16]. Balogu et al. [37] assessed the effect of 0.5T SMF on the microbial growth of Nono drinks, and the result illustrated that SMF can reduce the microbial growth rate and slow down the spoilage rate. In this experiment, compared with the MAP and SMF, the inhibitory effect of SMF combined with MAP on bacterial growth was more prominent. This demonstrated that the superposition of these two technologies could effectively enhance the inhibition effect. 

### 3.6. Effects of SMF Combined with MAP on pH, TBARS, TVB-N and Sensory

The overall pH showed a downtrend in all groups (Figure 3b). CO_2_ dissolution [38] or the production of organic acids (breakdown of carbohydrates by microorganisms) has been linked to promoting pH decline in meats [39,40]. In this study, the pH of the control, SMF, and MAP groups decreased with storage time, with the least values detected in the S + M group (*p* < 0.05). This may be attributed to the superposition of these two methods affecting the ability of microorganisms to decompose and produce acid.

Notably, the TVB-N and TBARS have been commonly explored as integral spoilage indicators [18]. Although the TVB-N content of the S + M group was lower than the other groups from 0 to 48 h (Figure 4a,b), no variation difference was observed between the groups at 80 h (*p* > 0.05). At 80 h, an uptrend in TBARS was observed in the control group compared to the others (*p* < 0.05). The above results indicated that, although the three treatments (SMF, MAP, and S + M) did not influence protein and lipid oxidative stresses in the meatballs, they could effectively inhibit the formation of TBARS during storage. 

**Figure 5:**
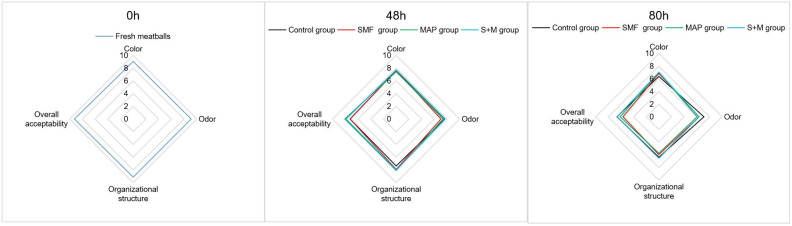
Effects of different treatments on the sensory perception of meatballs (*n* = 3).

The sensory characters (color, odor, organizational structure, and overall acceptability) of meatballs were determined (Figure 5). Specifically, the control group scored lowest in tissue structure, while the S + M group scored highest in odor and overall acceptability at 48 h. At 80 h, the S + M group scored higher on color, texture, and overall acceptability, indicating that S + M treatment did not significantly affect meatball sensory perception and can still maintain its sensory level to a certain extent. Hence, it was beneficial in maintaining the sensory attributes of the meatballs.

## 4. Conclusions

In this study, the BPS group demonstrated a 2.26-fold increase in volume compared to the spoilage samples stored at 15 °C, including a CO_2_ uptrend in the packs. The BPS meatballs, with *Klebsiella* spp. (46.19%) and *Escherichia* spp. (40.19%) as dominant genera had a distinct bacterial community from the spoilage group. Inoculation experiments confirmed that *K. pneumonia* isolates, as the gas-producing bacteria, primarily induced BPS occurrence in meatballs products. The synergy of SMF (5 mT) and MAP (40%CO_2_ + 60%N_2_) can significantly prevent an increase in pack volume and inhibit CO_2_ production. Additionally, compared with the control group, the synergistic treatment delayed bacterial growth, pH decline, inhibited oxidative stress, and did not impede the quality of meatballs. The results will provide meat scientists and processors insight into facilitating effective strategies to mitigate BPS occurrence in meatballs, thereby prolonging shelf life.

## Figures and Tables

**Table 1:** Scoring standard for sensory description test of meatballs.

Item	Sensory Description	Score
Color	Shiny and even flesh color	8–10
	Slightly shiny, uneven flesh color	4–7
	Uneven color, dull	1–3
Odor	Rich and pure, and the overall smell is harmonious	8–10
	Strong fragrance, no bad smell	4–7
	Unpleasant smell, the overall smell is not harmonious	1–3
Organizational structure	Smooth cut surface and dense structure	8–10
	Slightly rough-cut surface and loose structure	4–7
	Rough cut surface, loose structure	1–3
Overall acceptability	Appearance is highly acceptable and appetite is strong	8–10
	Appearance is acceptable, and appetite is slightly strong	4–7
	Unacceptable appearance, weak appetite	1–3

**Table 2:** Gas compositions and TVC in the spoilage (S) and BPS (B) groups during storage at 4 days. The different lower case letters (a,b) indicate significant differences between the spoilage (S) and BPS (B) groups (*p* < 0.05). The results were calculated as the means and standard errors and statistically analyzed using ANOVA (*n* = 3).

Index	S	B
O_2_ (%)	9.06 ± 1.98% ^a^	0.13 ± 0.03% ^b^
CO_2_ (%)	7.47 ± 1.27% ^b^	71.85 ± 0.65% ^a^
TVC (log CFU/g)	8.10 ± 0.20 ^a^	8.28 ± 0.05 ^a^

**Table 3:** Volume change rate and CO_2_ percentage in BPS groups after inoculation during storage at 4 days, and kinetic growth parameters of 35 *K. pneumoniae* strains. Results were calculated as mean and standard errors (*n* = 3).

Strains No.	Volume Increase	CO_2_ Percentage (%)	Lag	Y_max_
A2	2.27 ± 0.08	65.90 ± 0.90	4.361 ± 0.021	3.505 ± 0.005
A3	2.17 ± 0.09	65.53 ± 0.95	4.512 ± 0.139	3.539 ± 0.005
A12	2.33 ± 0.13	66.63 ± 1.13	4.074 ± 0.245	3.502 ± 0.015
A35	2.03 ± 0.17	65.17 ± 1.88	4.395 ± 0.227	3.549 ± 0.011
A40	2.36 ± 0.03	67.27 ± 2.39	4.885 ± 0.320	3.531 ± 0.018
A41	2.36 ± 0.05	64.53 ± 1.56	4.310 ± 0.213	3.548 ± 0.005
A43	2.42 ± 0.06	63.87 ± 2.19	4.285 ± 0.115	3.556 ± 0.010
A45	2.03 ± 0.02	56.93 ± 1.44	4.613 ± 0.288	3.539 ± 0.006
A46	2.32 ± 0.03	64.07 ± 1.79	4.703 ± 0.589	3.538 ± 0.006
A49	2.13 ± 0.05	64.03 ± 0.86	4.789 ± 0.135	3.555 ± 0.002
B5	2.62 ± 0.09	70.80 ± 1.04	3.502 ± 0.143	3.624 ± 0.009
B8	2.15 ± 0.03	64.20 ± 0.70	4.150 ± 0.103	3.226 ± 0.068
B9	2.36 ± 0.08	65.27 ± 1.60	4.538 ± 0.098	3.562 ± 0.007
B15	2.33 ± 0.06	66.57 ± 1.08	4.329 ± 0.100	3.545 ± 0.006
B16	2.35 ± 0.00	65.33 ± 0.84	3.573 ± 0.290	3.380 ± 0.006
B18	2.16 ± 0.00	67.73 ± 1.20	4.363 ± 0.190	3.564 ± 0.015
B19	2.13 ± 0.03	64.43 ± 1.79	4.659 ± 0.101	3.559 ± 0.016
B21	2.39 ± 0.09	63.60 ± 0.80	4.529 ± 0.019	3.556 ± 0.004
B22	2.35 ± 0.01	63.17 ± 0.43	4.935 ± 0.390	3.538 ± 0.015
B24	2.11 ± 0.03	61.83 ± 1.47	3.945 ± 0.067	3.252 ± 0.027
B25	2.18 ± 0.01	61.93 ± 0.30	4.568 ± 0.159	3.505 ± 0.007
B28	2.09 ± 0.04	62.13 ± 0.60	4.841 ± 0.220	3.517 ± 0.011
B29	2.12 ± 0.01	68.87 ± 0.80	4.413 ± 0.103	3.236 ± 0.007
B33	2.33 ± 0.17	65.43 ± 1.52	4.224 ± 0.523	3.513 ± 0.006
C3	2.28 ± 0.06	65.63 ± 1.45	4.223 ± 0.128	3.506 ± 0.012
C6	2.24 ± 0.10	64.87 ± 1.94	3.647 ± 0.172	3.358 ± 0.013
C18	2.59 ± 0.09	64.23 ± 0.47	4.468 ± 0.184	3.490 ± 0.014
C19	2.74 ± 0.16	63.90 ± 0.075	5.157 ± 0.408	3.512 ± 0.002
C21	2.66 ± 0.13	62.20 ± 0.47	4.757 ± 0.155	3.467 ± 0.020
C23	2.10 ± 0.06	62.50 ± 1.45	3.630 ± 0.092	3.291 ± 0.006
C24	2.31 ± 0.08	61.10 ± 0.21	3.633 ± 0.029	3.304 ± 0.003
C25	2.15 ± 0.15	63.17 ± 0.67	4.655 ± 0.181	3.519 ± 0.028
C36	2.38 ± 0.09	62.77 ± 0.96	4.572 ± 0.323	3.505 ± 0.008
C37	2.15 ± 0.03	64.07 ± 1.90	3.695 ± 0.215	3.316 ± 0.002
C41	2.10 ± 0.05	61.57 ± 0.87	4.681 ± 0.192	3.517 ± 0.005

**Table 4:** Physiological and biochemical analysis of 35 *K. pneumoniae* strains.

Property	Results	Property	Results
Gram stain	−	Urease	+
Oxidase	−	Citrate utilization	+
H_2_S	−	Malonate utilization	+
Ornithine decarboxylase	−	Rhamnose fermentation	+
MR test	−	Inositol fermentation	+
VP test	+	Glucose fermentation	+

Note: − negative; + positive.

## Data Availability

Data is contained within the article.

## References

[1] Reid R., Fanning S., Whyte P., Kerry J., Bolton D. (2017). An investigation of the effect of rapid slurry chilling on blown pack spoilage of vacuum-packaged beef primals. Lett. Appl. Microbiol..

[2] Silva A.R., Paulo É.N., Sant’Ana A.S., Chaves R.D., Massaguer P.R. (2011). Involvement of *Clostridium gasigenes* and *C. algidicarnis* in “blown pack” spoilage of Brazilian vacuum-packed beef. Int. J. Food Microbiol..

[3] Broda D.M., Saul D.J., Lawson P.A., Bell R.G., Musgrave D.R. (2000). *Clostridium gasigenes* sp. nov., a psychrophile causing spoilage of vacuum-packed meat. Int. J. Syst. Evol. Microbiol..

[4] Dainty R.H., Edwards R.A., Hibbard C.M. (1989). Spoilage of vacuum-packed beef by a *clostridium* sp.. J. Sci. Food Agric..

[5] Kalchayanand N., Ray B., Field R.A., Johnson M.C. (1989). Spoilage of vacuum-packaged refrigerated beef by *Clostridium*. J. Food Protect..

[6] Zhang P., Ward P., Mcmullen L.M., Yang X. (2020). A case of “blown pack” spoilage of vacuum-packaged pork likely associated with *Clostridium estertheticum* in Canada. Lett. Appl. Microbiol..

[7] Húngaro H.M., Caturla M.Y.R., Horita C.N., Furtado M.M., Sant’Ana A.S. (2016). Blown pack spoilage in vacuum-packaged meat: A review on clostridia as causative agents, sources, detection methods, contributing factors and mitigation strategies. Trends Food Sci. Technol..

[8] Schuster J.A., Klingl A., Vogel R.F., Ehrmann M.A. (2019). Polyphasic characterization of two novel *Lactobacillus* spp. isolated from blown salami packages: Description of *Lactobacillus halodurans* sp. nov. and *Lactobacillus salsicarnum* sp. nov. Syst. Appl. Microbiol..

[9] Pinheiro R.S.B., Jorge A.M., Yokoo M.J. (2010). Correlações entre medidas determinadas in vivo por ultrassom e na carcaça de ovelhas de descarte. Rev. Bras. Zootec..

[10] Broda D.M. (2007). The effect of peroxyacetic acid-based sanitizer, heat and ultrasonic waves on the survival of *Clostridium estertheticum* spores in vitro. Lett. Appl. Microbiol..

[11] Boerema J.A., Broda D.M., Penney N., Brightwell G. (2007). Influence of peroxyacetic acid-based carcass rinse on the onset of “blown pack” spoilage in artificially inoculated vacuum-packed chilled beef. J. Food Prot..

[12] Adam K.H., Flint S.H., Brightwell G. (2013). Reduction of spoilage of chilled vacuum-packed lamb by psychrotolerant clostridia. Meat Sci..

[13] Hashish A.H., El-Missiry M.A., Abdelkader H.I., Abou-Saleh R.H. (2008). Assessment of biological changes of continuous whole body exposure to static magnetic field and extremely low frequency electromagnetic fields in mice. Ecotoxicol. Environ. Saf..

[14] Albuquerque W., Costa R., Fernandes T., Porto A. (2016). Evidences of the static magnetic field influence on cellular systems. Prog. Biophys. Mol. Biol..

[15] Filipič J., Kraigher B., Tepuš B., Kokol V., Mandic-Mulec I. (2012). Effects of low-density static magnetic fields on the growth and activities of wastewater bacteria *Escherichia coli* and *Pseudomonas putida*. Bioresour. Technol..

[16] Ji W., Huang H., Deng A., Pan C. (2009). Effects of static magnetic fields on *Escherichia coli*. Micron.

[17] Rossaint S., Klausmann S., Kreyenschmidt J. (2015). Effect of high-oxygen and oxygen-free modified atmosphere packaging on the spoilage process of poultry breast fillets. Poult. Sci..

[18] Li R., Cai L., Gao T., Li C., Zhou G., Ye K. (2020). Comparing the quality characteristics and bacterial communities in meatballs with or without blown pack spoilage. LWT.

[19] Huang L. (2014). IPMP 2013—A comprehensive data analysis tool for predictive microbiology. Int. J. Food Microbiol..

[20] Broda D.M., Delacy K.M., Bell R.G., Braggins T.J., Cook R.L. (1996). Psychrotrophic *Clostridium* spp. associated with “blown pack” spoilage of chilled vacuum-packed red meats and dog rolls in gas-impermeable plastic casings. Int. J. Food Microbiol..

[21] Chaves R.D., Silva A.R., SantAna A.S., Campana F.B., Massaguer P.R. (2012). Gas-producing and spoilage potential of Enterobacteriaceae and lactic acid bacteria isolated from chilled vacuum-packaged beef. Int. J. Food Sci. Technol..

[22] Massa S., Gardini F., Sinigaglia M., Guerzoni M.E. (1992). *Klebsiella pneumoniae* as a spoilage organism in mozzarella Cheese. J. Dairy Sci..

[23] De Vos P. (2009). Bergey’s Manual of Systematic Bacteriology.

[24] Krishnamoorthy R., Athinarayanan J., Periyasamy V.S., Alshuniaber M.A., Alshammari G., Hakeem M.J., Ahmed M.A., Alshatwi A.A. (2022). Antibacterial Mechanisms of Zinc Oxide Nanoparticle against Bacterial Food Pathogens Resistant to Beta-Lactam Antibiotics. Molecules.

[25] Guo Y., Zhou H., Qin L., Pang Z., Qin T., Ren H., Pan Z., Zhou J. (2016). Frequency, antimicrobial resistance and genetic diversity of *Klebsiella pneumoniae* in food samples. PLoS ONE.

[26] Zhang S., Yang G., Ye Q., Wu Q., Zhang J., Huang Y. (2018). Phenotypic and genotypic characterization of *Klebsiella pneumoniae* isolated from retail foods in China. Front. Microbiol..

[27] Abdel-Rhman S.H. (2020). Characterization of β-lactam resistance in *K. pneumoniae* associated with ready-to-eat processed meat in Egypt. PLoS ONE.

[28] Calbo E., Freixas N., Xercavins M., Riera M., Nicolas C., Monistrol O. (2011). Foodborne nosocomial outbreak of SHV1 and CTX-M-15-producing *Klebsiella pneumoniae*: Epidemiology and control. Clin. Infect. Dis..

[29] Srinivasan S., Feng S., Lin Y. (2012). Dissolved carbon dioxide concentration profiles during very-high-gravity ethanol fermentation. Biochem. Eng. J..

[30] Mills J., Horváth K.M., Brightwell G. (2018). Antimicrobial effect of different peroxyacetic acid and hydrogen peroxide formats against spores of *Clostridium estertheticum*. Meat Sci..

[31] Dixon Nm K.D. (1989). A Review:The inhibition by CO_2_ of the growth and metabolism of microorganisms. J. Appl. Bacteriol..

[32] Arvanitoyannis I.S., Stratakos A.C. (2012). Application of modified atmosphere packaging and active/smart technologies to red meat and poultry: A Review. Food Bioprocess Technol..

[33] Bubanja I.N., Lončarević B., Lješević M., Beškoski V., Gojgić-Cvijović G., Velikić Z., Stanisavljev D. (2019). The influence of low-frequency magnetic field regions on the *Saccharomyces cerevisiae* respiration and growth. Chem. Eng. Process.—Process Intensif..

[34] Cao J., Liu W., Mei J., Xie J. (2021). Effect of locust bean gum-sodium alginate coatings combined with high CO_2_ modified atmosphere packaging on the quality of turbot (*Scophthalmus maximus*) during refrigerated storage. Polymers.

[35] Wang Q., Chen Q., Xu J., Sun F., Liu H., Kong B. (2022). Effects of modified atmosphere packaging with various CO_2_ concentrations on the bacterial community and shelf-life of smoked chicken Legs. Foods.

[36] Guo Y., Huang J., Sun X., Lu Q., Huang M., Zhou G. (2018). Effect of normal and modified atmosphere packaging on shelf life of roast chicken meat. J. Food Saf..

[37] Balogu T.V., Attansey C.R. (2017). Effect of static magnetic field on microbial growth kinetics and physiochemical properties of nono (fermented milk drink). J. Microbiol. Biotechnol. Food Sci..

[38] Gokoglu N., Yerlikaya P., Uran H., Topuz O.K. (2010). The effect of modified atmosphere packaging on the quality and shelf life of frankfurter type-sausages. J. Food Qual..

[39] Viana B.I., Domenici M.O., Jorge E.V., Vieira F.J., Lopes F.M., Cangussu A.S.R., Sobrinho E.M. (2014). Growth conditions of clostridium perfringens type B for production of toxins used to obtain veterinary vaccines. Bioprocess Biosyst. Eng..

[40] Luong N.M., Coroller L., Zagorec M., Moriceau N., Anthoine V., Guillou S., Membré J.M. (2022). A Bayesian Approach to Describe and Simulate the pH Evolution of Fresh Meat Products Depending on the Preservation Conditions. Foods.

